# Supplementary material for: The interactome of intact mitochondria by cross-linking mass spectrometry provides evidence for coexisting respiratory supercomplexes
Source: Mol Cell Proteomics. 2017 Dec 8;17(2):216–32. doi: 10.1074/mcp.RA117.000470 (PMC5795388; doi:10.1074/mcp.RA117.000470)
Supplement: Supplemental Data [file supp_RA117.000470_133922_0_supp_23978_fzffwf.zip › spectra_annotation/mito_spectra_annotation/456-1-6-1-2-1.pdf]

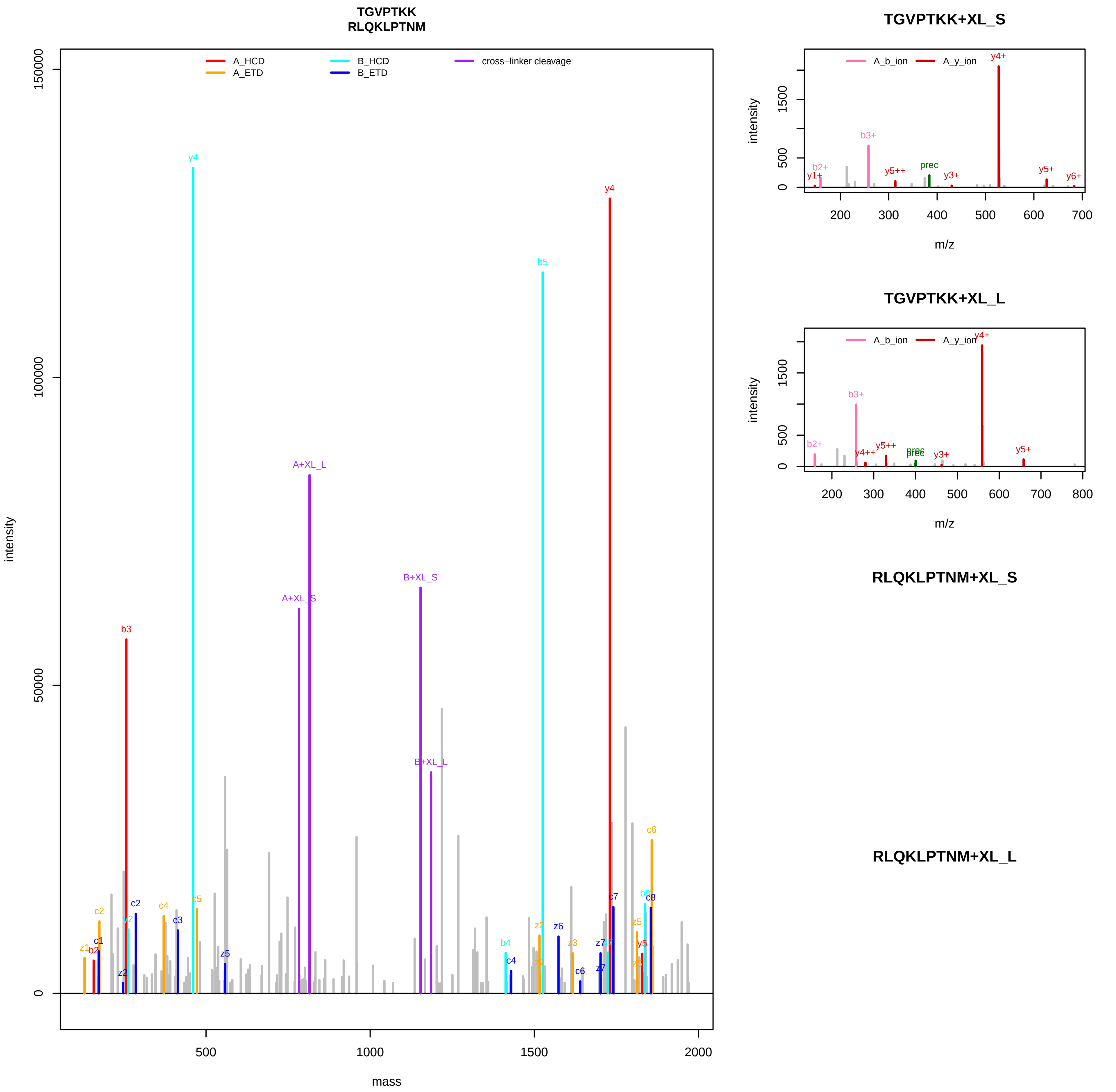
TGVPTKK
RLQKLPTNM
A_HCD
A_ETD
B_HCD
B_ETD
cross−linker cleavage
intensity
mass
0
50000
100000
150000
500
1000
1500
2000
y4
b5
A+XL_L
A+XL_S
B+XL_S
B+XL_L
b3
c6
c7
c8
b8
z6
z3
z5
y5
c2
c4
c5
c3
c1
b2
z1
z2
z5
b4
c4
c6
z7
TGVPTKK+XL_S
A_b_ion
A_y_ion
y4+
b3+
b2+
y1+
y5++
prec
y3+
y5+
y6+
m/z
TGVPTKK+XL_L
y4++
RLQKLPTNM+XL_S
RLQKLPTNM+XL_L
